# Aptamer modified magnetic nanoparticles for the determination of the allergenic protein α-lactalbumin in food samples

**DOI:** 10.1007/s00604-026-07936-5

**Published:** 2026-03-03

**Authors:** Natalia Piqueras-García, Raúl Mínguez-Peláez, María Vergara-Barberán, José Manuel Herrero-Martínez, María Jesús Lerma-García

**Affiliations:** Department of Analytical Chemistry, University of Valencia, Avda. Vicent Andrés Estellés 19, 46100 Burjassot, Valencia, Spain

**Keywords:** α-Lactalbumin, Magnetic solid-phase extraction, HPLC-DAD, Aptamer, Selective sorbent, Vinylated magnetic nanoparticles

## Abstract

Aptamer-modified magnetic nanoparticles (Apt-MNPs) were synthetized and used as a magnetic solid-phase extraction (MSPE) sorbent for the selective isolation of α-lactalbumin (α-LA), an allergenic protein from cow’s milk. For this purpose, a thiol-terminated Apt was immobilized onto the surface of vinyl-functionalized MNPs via "thiol-ene" click chemistry reaction. The resulting Apt-modified MNPs were characterized and several parameters that affect the Apt-based MSPE efficiency were investigated. Under the optimal conditions, the developed Apt-based MSPE protocol was applied to the determination of α-LA in different foodstuffs (dairy-free products and those with declared milk content), yielding satisfactory recoveries (80.0–96.9%), good precision (RSD < 5.5%), and a low limit of detection (0.04 μg·mL⁻^1^). Moreover, the Apt-based sorbent proved to be highly affordable, with a cost of less than 4.2 € per sorbent, further supporting its suitability for routine analysis.

**Graphical Abstract:**

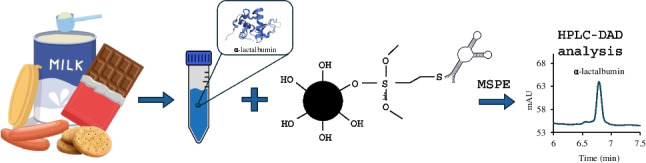

**Supplementary Information:**

The online version contains supplementary material available at 10.1007/s00604-026-07936-5.

## Introduction

One of the most prevalent childhood food allergies is to cow’s milk proteins [1], with α-lactalbumin (α-LA) being a relevant allergenic whey protein due to its high sensitization rate and widespread use as a nutritional ingredient [2]. As no curative treatment is currently available, management relies on strict avoidance of allergenic foods, making the development of sensitive, efficient, and reliable analytical methods essential to prevent inadvertent ingestion of proteins such as α-LA and to ensure compliance with regulatory thresholds [3].

Several analytical techniques are available for detecting allergenic proteins in food matrices, including enzyme-linked immunosorbent assays (ELISA) [4, 5], polymerase chain reaction (PCR) [6], and high-performance liquid chromatography (HPLC), often coupled to mass spectrometry (MS) [7–9]. However, these methods present limitations: ELISA suffers from high costs, antibody thermal instability, and cross-reactivity, which can compromise accuracy, especially in processed foods. On the other hand, HPLC–MS offers high sensitivity and selectivity but requires expensive instrumentation, long analysis times and highly trained personnel, limiting its routine applicability [8, 9]. Chromatographic approaches using more economical detectors (e.g., UV–Vis or fluorescence detection) remain attractive alternatives; however, their performance strongly depends on efficient and selective sample preparation. Current sample preparation strategies are often labor-intensive, non-selective and insufficient to cope with matrix interferences, which can reduce sensitivity and reproducibility when analyzing trace levels of allergenic proteins [10, 11]. To overcome these analytical challenges, Aptamers (Apts) have emerged as alternative recognition ligands due to their high affinity, selectivity, chemical robustness, and ease of functional modification [12, 13], which can be developed against a broad range of targets. In this regard, Apts have been successfully implemented as affinity ligands in sample preparation [12, 14, 15] on different solid supports, including metal–organic frameworks [16], carbon-based materials [17], stir bars [18], cellulose papers [19, 20], melamine sponges [21], polypropylene tubes [22], and fibers [23], among others. Among these applications, the integration of Apts with magnetic materials has gained particular attention for the development of selective and efficient sample preparation strategies [24], concretely Apt-functionalized magnetic nanoparticles (MNPs) have been reported for the selective extraction of herbicides [25], toxins [26], antibiotics [27], and polycyclic aromatic hydrocarbons [28], among others; however, the analysis of macromolecules using Apt-based MSPE sorbents remains scarcely explored. Despite this limited number of extraction-oriented studies, Apt–magnetic particle platforms have been employed for the selective recognition of macromolecular targets in sensing applications. In particular, optical apta-assays based on magnetic beads or NPs have enabled the detection and quantification of protein allergens such as β-conglutin, gliadin, Ara h1, and lysozyme in complex food matrices [29]. These studies highlight the high affinity and selectivity of Apts toward large biomolecules and support their potential applicability in macromolecular analysis. Nevertheless, the translation of Apt–magnetic platforms from sensing to MSPE-based extraction faces several challenges, including the structural complexity of macromolecules, potential conformational changes of Apt upon immobilization on MNPs, difficulties associated with the efficient elution of intact macromolecules, limited reusability due to loss/degradation of Apt, and the limited availability of well-characterized Apt sequences for many relevant macromolecular targets. These limitations partly explain the current scarcity of Apt-based MSPE approaches for macromolecular analysis and underscore the need for further methodological development.

To address this gap, the present work develops and evaluates an Apt-functionalized magnetic sorbent for the selective extraction and determination of α-LA in milk-free and milk-containing food samples. To obtain the sorbent, a thiol-terminated Apt was covalently attached to the vinilyzed surface of MNPs, and the resulting Apt-MSPE sorbent was characterized and evaluated for α-LA extraction performance.

## Experimental

### Reagents and materials

2,2'-azobis(2-methylpropionitrile) (AIBN), vinyltrimethoxysilane (VTMS), tris(hydroxymethyl)aminomethane (Tris), tris(2-carboxyethyl)phosphine (TCEP), trisodium citrate, imidazole, glycine, and acetic acid (glacial) 100% were from Sigma-Aldrich (Darmstadt, Germany). Potassium chloride was from Scharlau (Barcelona, Spain). Petroleum ether and iron (II) chloride tetrahydrate were provided by VWR Chemicals (Barcelona, Spain). Ethanol and acetonitrile (ACN) were obtained from PanReac AppliChem (Barcelona, Spain). Iron (III) chloride hexahydrate was procured from Probus (Barcelona, Spain). Magnesium chloride hexahydrate, ammonia solution 25%, urea, hydrochloric acid 37%, sodium chloride, and sodium hydroxide were sourced from Labkem (Barcelona, Spain). Trifluoroacetic acid (TFA) 99% was obtained from Thermo Scientific (Valencia, Spain).

The protein used for the assays was α-LA supplied by Sigma-Aldrich. A 1 mg·mL^−1^ α-LA solution was prepared in water, aliquoted, and stored at −20 ºC. Aliquots were defrosted before use, and working standard solutions were prepared daily by dilution in the appropriate buffer solution. Protein-containing assays were carried out using 2 mL LoBind Eppendorfs (Thermo Fisher Scientific, Massachusetts, USA).

The Apt binding buffer (BB) was composed by 50 mM Tris–HCl, 100 mM NaCl, 5 mM KCl, and 1 mM MgCl_2_ at pH = 7.4 [2], which was stored in fridge until use. The α-LA-specific Apt modified at its 5’ end with a thiol group and a C6 spacer arm (5’-/5ThioMC6-D/AGC AGC ACA GAG GTC AGA TGG TGC TGC GAA-3’) [2] was synthesized and purified by Integrated DNA Technologies (Coralville, IA, United States). This Apt was dissolved in nuclease-free water (VWR, Barcelona, Spain) resulting in an Apt final concentration of 100 µmol·L^−1^.

The food samples used in this work were purchased from a local supermarket, and included two dairy-free food samples (a hydrolyzed hypoallergenic infant formula (milk substitute) and sausages), one sample with declared milk trace levels (biscuits), and two samples with label declared milk (infant formula and milk chocolate).

### Instrumentation

FTIR spectra of MNPs were recorded using a FTIR Cary 630 instrument from Agilent Technologies (Waldbronn, Germany) equipped with an attenuated total reflection (ATR) accessory. All spectra were measured at room temperature with a resolution of 4 cm^−1^. The FTIR spectra were obtained from 4000 to 500 cm^−1^ in absorbance mode.

The morphology of the MNPs was characterized by high-resolution transmission electron microscopy (HRTEM) using a Tecnai microscope from FEI (United States). X-ray diffraction (XRD) patterns of magnetic materials were obtained using a D8 Advance A25 X-ray diffractometer (Bruker, Berlin, Germany). Nitrogen adsorption–desorption isotherms were measured using a S5 Micromeritics ASAP2020 instrument (Norcross, USA) at 77 K, and the specific surface area was calculated using the Brunauer-Emmet-Teller (BET) mathematical model. Surface analysis was also carried out by X-ray photoelectron spectroscopy (XPS) using a K-Alpha™ XPS system equipped with a monochromatic Al K(alpha) source (1486.6 eV).

The determination of phosphorous content in vinylated MNPs and Apt@MNPs was done by using a 7900 inductively coupled plasma mass spectrometer (ICP-MS) from Agilent Technologies. For this purpose, P content was quantified by measuring the signal ratio between ^31^P and isotope ^72^Ge (internal standard). The calibration curve ranged from 0 to 2000 μg·L^−1^ of P.

α-LA concentration was determined using a 1100 Series HPLC system (Agilent Technologies), equipped with a binary pump, degasser, autosampler, and diode array detector (DAD). The chromatographic system was controlled by an OpenLAB CDS LC ChemStation from Agilent (B.04.03).

### Preparation of Apt-vinylated MNPs

To carry out the synthesis of the Apt-functionalized MNPs, a three-step procedure was performed based on a previous work [25]. First, Fe_3_O_4_ MNPs were synthesized by introducing 180 mL of an aqueous solution containing 11.2 mmol FeCl_3_·6H_2_O and 5.6 mmol FeCl_2_·4H_2_O into a three-neck round-bottom flask. This solution was heated up to 50 °C in a silicon oil bath under stirring, after which 15 mL of ammonia solution was added. The mixture was left at 50 °C for 30 min, and then the temperature was raised up to 90 °C and held for another 30 min. The synthesis was carried out under a nitrogen atmosphere. Once the MNPs were synthesized, they were collected with an external magnet and washed with water (to remove excess of ammonia) and with ethanol; the remaining MNPs were placed in an oven at 60 ºC overnight.

The second step of the synthesis involved MNPs vinylation. For this end, 0.5 g MNPs were added to 125 mL of ethanol in a three-neck round-bottom flask and were sonicated for 15 min. The resulting dispersion was purged with nitrogen and 4 mL of ammonia solution and 4 mL VTMS were added, being the resulting mixture left overnight under continuous stirring. Once the vinylated MNPs (VMNPs) were obtained, they were washed with ethanol and dried in an oven at 60 °C overnight.

The final step of the synthesis consisted in the functionalization of the VMNPs with the specific α-LA Apt, which was previously reduced as follows: 50 μL of the 100 µM thiol-modified Apt was mixed with 20 µL of 50 mM TCEP, vortexed, and incubated in the dark for 2 h to reduce Apt disulfide bonds to free thiol groups. Then, the solution was heated at 95 °C for 10 min and cooled to 4 °C for another 10 min to promote Apt folding. Subsequently, the ‘thiol-ene’ click reaction was performed by adding 0.5 mL of 0.9% (v/w) AIBN solution prepared in ACN:H_2_O (50:50 v/v) to 25 mg of VMNPs, followed by the addition of 5 μL of the reduced and folded Apt solution. The resulting mixture was vortexed for 1 min and stirred at 900 rpm in a thermo-shaker at 55 °C for 5 h. The resulting Apt@VMNPs were collected using a magnet, washed three times with water, and resuspended in 1 mL of BB until use.

In order to minimize non-specific interactions between α-LA (or other proteins) and Apt@VMNPs, the surface was blocked with trisodium citrate to provide a more hydrophilic and less adsorptive interface [30, 31]. For this purpose, 25 mg of Apt@VMNPs were mixed with 2 mL of 0.5 M trisodium citrate for 1 h at 95 ºC and 1500 rpm. Then, the resulting Apt@cit-VMNPs were washed with water and stored at 4 ºC until use.

### Protein extraction from food samples

Prior to protein extraction, non-powdered foodstuffs (two chocolate samples, biscuits and sausages) were ground and defatted via Soxhlet extraction with petroleum ether for 1 h. Subsequently, proteins extraction of all samples was performed based on a previous study [18]. Briefly, 1 g of each sample was weighed into a 15 mL Falcon tube, and 10 mL of 50 mM Tris buffer at pH 7.4 was added. The dispersion was heated in a water bath at 40 °C for 30 min, and then centrifuged at 3500 rpm for 15 min. The supernatant was acidified to pH 4.6 with 1 M HCl and left to stand for 20 min at room temperature to precipitate caseins. They were separated from the supernatant by centrifugation at 3500 rpm and 4 °C for 10 min. Finally, the resulting solutions were neutralized to pH 7.4 with 1 M NaOH and stored in a refrigerator at 4 ºC. In the case of dairy-free and declared milk trace levels samples, spiked extracts were prepared by adding an appropriate volume of 1000 μg·mL⁻1 α-LA standard solution to obtain final concentrations of 0.4 µg and 1.0 µg α-LA per mL (4 and 10 mg·kg^−1^). For samples with label declared milk, the corresponding sample extract was 1:100 (v/v) diluted, and then spiked with an appropriate volume of 1000 μg·mL⁻1 α-LA standard solution to obtain a final concentration of 1.0 µg α-LA per mL (1 mg·g^−1^).

### Apt-MSPE protocol

α-LA extraction was carried out by mixing 25 mg Apt@cit-VMNPs with 2 mL α-LA 1 µg·mL^−1^ standard solution prepared in 50 mM Tris buffer at pH 7.4. Subsequently, the suspension was placed in a thermo-shaker at 900 rpm for 10 min at 25 ºC. After that, the sorbent was separated from the solution using a magnet and was washed with water to remove non-retained compounds. Elution was performed by adding 1 mL of 4 M imidazole to the isolated Apt@cit-VMNPs, being the suspension shaken at 900 rpm for 10 min at 50 °C. Next, a regeneration step was carried out by washing the Apt@cit-VMNPs with water and then mixed with 2 mL of 0.5 M trisodium citrate for 1 h at 95 ºC and 1500 rpm. Finally, the magnetic sorbent was washed with 1 mL of 50 mM Tris buffer at pH 7.4 for 1 min at room temperature and 900 rpm.

During the extraction optimization studies with α-LA standard, all fractions were collected and analyzed by HPLC–UV to evaluate α-LA retention and recovery. Retention was calculated as the ratio of α-LA concentration in the remaining solution (after contact with Apt@cit-VMNPs) to its concentration in the initial loading solution. On the other hand, recovery was calculated similarly but considering the α-LA concentration in the elution fraction and taking into account the percentage of protein initially retained. After Apt-MSPE protocol optimization, it was applied to the different food extracts. All experiments were carried out by triplicate.

### Determination of α-LA concentration

Chromatographic analysis of α-LA in all the fractions obtained in the previous section was accomplished using a C4 XBridge® BEH column (300 Å, 4.6 × 150 mm, 3.5 μm) (Waters, Milford, MA, USA) at room temperature. The injection volume was 100 µL, and the detection wavelength was 220 nm. The mobile phases used were the following: (A) water with 0.1% TFA and (B) ACN with 0.1% TFA, using a flow rate of 1 mL·min^−1^. The elution gradient used was as follows 35% B from 0–2 min, followed by an increase to 45% B from 2–6 min, and maintained at 45% B from 6–8 min. A re-equilibration step was then applied for 5 min to reach the initial conditions.

## Results and discussion

### Apt@cit-VMNP characterization

**Fig. 1:**
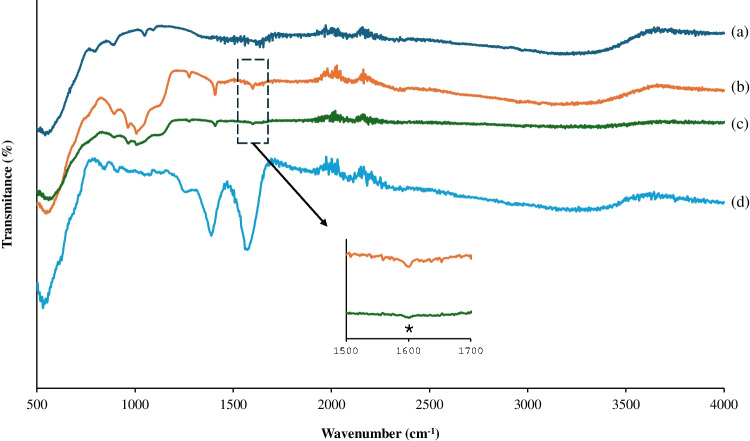
FTIR spectra of MNPs (**a**), VMNPs (**b**), Apt@VMNPs (**c**), and Apt@cit-VMNPs (**d**)

In order to confirm the successful synthesis of Apt@cit-VMNPs, the different steps (MNPs vinylation, Apt immobilization onto the surface of VMNPs, and Apt@VMNPs blocking with trisodium citrate) were corroborated by FTIR (see Fig. 1**)**. The presence of an absorption band at 550 cm^−1^ in all spectra, which corresponds to the Fe–O bond characteristic of the magnetite phase, confirms the correct synthesis of the MNPs. Regarding the vinylation step (Fig. 1b), a band at 1050 cm^−1^, attributed to the Si–O-Si asymmetric stretching bonds, jointly with two bands at 1407 and 1620 cm^−1^, assigned to the CH asymmetric bending vibrations and C = C stretching vibrations on Si-CH = CH_2_, confirm the successful vinylation of the MNPs. After functionalisation of the VMNPs with the Apt (Fig. 1c), an absorbance decrease of the band at 1620 cm^−1^ is observed, confirming the anchoring of the Apt to the VMNPs through the ‘thiol-ene’ click reaction. Furthermore, Fig. 1d shows the spectra of Apt@cit-VMNPs, where two new absorption bands appear at 1388 and 1560 cm^−1^, attributed to the symmetric and asymmetric stretching vibrations of the carboxylate groups of trisodium citrate [32], respectively, confirming the successful binding of citrate to the surface of Apt@VMNP. Also, the increase in intensity of the broad band around 3000–3400 cm⁻^1^ suggests enhanced surface hydrophilicity after citrate coating, which contributes to improved colloidal stability of the magnetic sorbent.

**Fig. 2:**
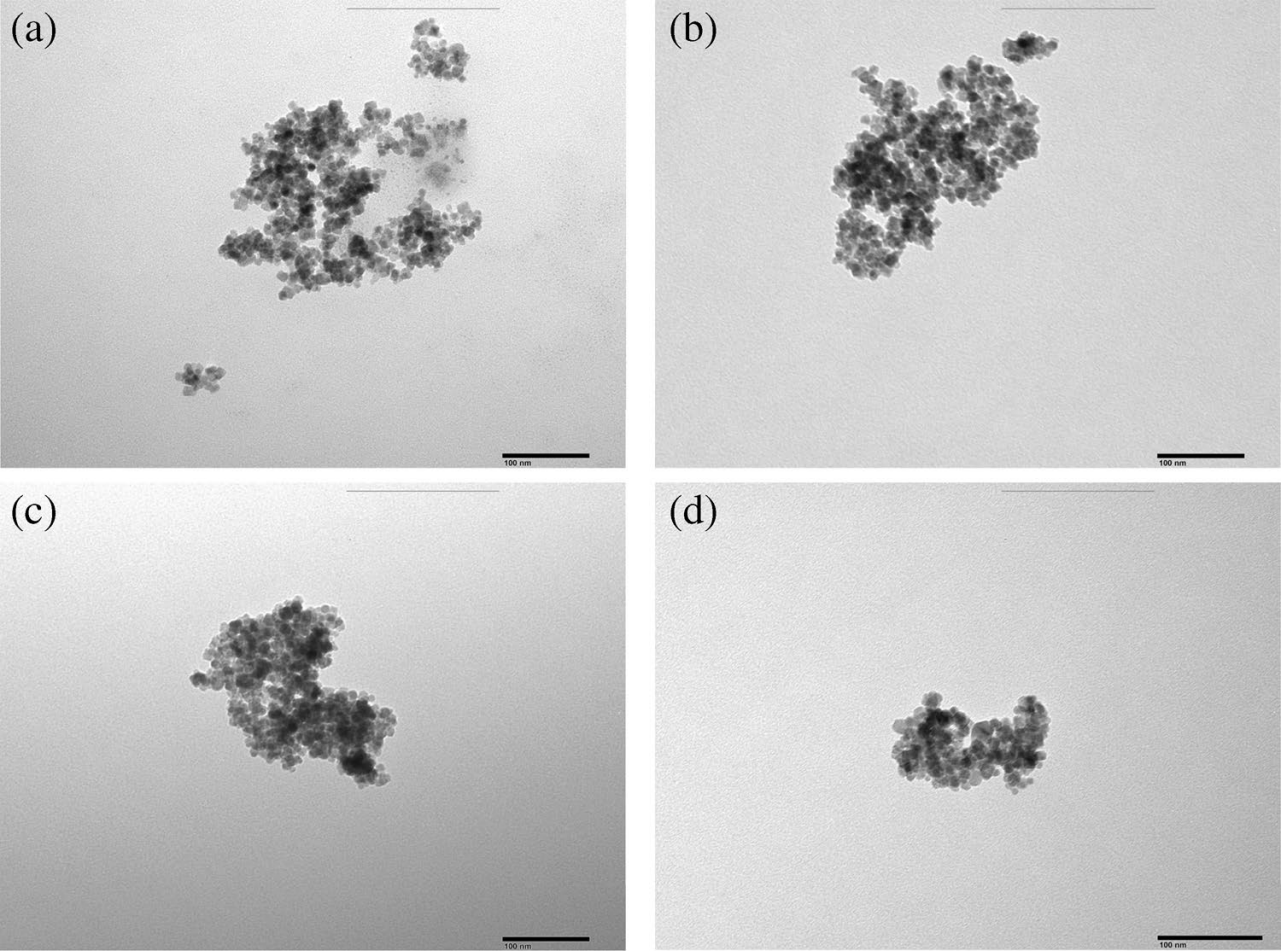
TEM images of MNPs (**a**), VMNPs (**b**), Apt@VMNPs (**c**) and Apt@cit-VMNPs (**d**)

Next, the morphological structure of the Apt@cit-VMNPs was characterized using TEM (Fig. 2). As shown in this figure, MNPs, VMNPs, Apt@VMNPs and Apt@cit-VMNPs showed similar particle sizes, with average diameters comprised between 14–18 nm, which is in accordance with previous studies [25]. XRD patterns of MNPs. VMNPs and Apt@cit-VMNPs were recorded and comparatively displayed in Fig. S1. As observed, the pattern of both VMNPs and Apt@cit-VMNPs showed the same characteristic peaks of bare Fe_3_O_4_ NPs, which indicates that the functionalization does not affect the core magnetite. The thin amorphous organic layer formed after Apt immobilization did not produce additional detectable diffraction peaks; therefore, no additional crystalline phases were observed in the diffractogram. The diffraction peaks located at 2θ = 30.1°, 35.6°, 43.4°, 53.6°, 56.9° and 62.7°, can be assigned to the (220), (311), (400), (422), (511) and (440) crystal planes of Fe_3_O_4_, respectively, confirming the preservation of characteristic cubic spinel lattice of magnetite after functionalization. Also, BET surface analysis revealed no significant change in the specific surface area (~ 107 m^2^g⁻^1^) after Apt immobilization, confirming that the modification is confined to MNPs’ surface and did not alter the textural properties of the magnetic core (see Fig. S2). Further characterization was conducted by XPS to investigate the surface elemental composition and to prove Apt immobilization. Fig. S3a and b illustrates the XPS survey spectra of MNPs before and after Apt functionalization. As observed, the main constituent elements of MNPs were Fe, O and C, with characteristic binding energy peaks located at 710–725 eV, 530.1 eV and 285.1 eV, respectively. After functionalization with Apt, additional peaks corresponding to Si, N and P appeared at approximately 102, 400 and 133 eV, respectively, as clearly evidenced in the high-resolution core-level spectra of Apt@cit-VMNPs (Fig. S3c-h). Thus, the Si 2p signal is consistent with a siloxane network originating from the silane coupling layer, while the N 1 s spectrum exhibits components characteristic of amine/amide and nucleobase-like nitrogen environments. Furthermore, the P 2p peak at ~ 133 eV corresponds to phosphate groups, providing direct evidence of the presence of the Apt backbone on the MNPs’ surface. Thereby, the XPS analysis confirmed the successful Apt immobilization onto MNPs. To complete the characterization study, ICP-MS analysis was also carried out before and after attachment of Apt onto VMNPs. Thus, Apt@VMNPs showed a phosphorus content of 0.0089 ± 0.0003%, which was considerably higher than that of the VMNPs (< 0.0005%). The residual phosphorus content detected in this control sample can be attributed to impurities in the reagents and water used during the MNPs functionalization process. These results further support the successful immobilization of the Apt onto the MNPs.

### Selection of optimal MSPE conditions

**Fig. 3:**
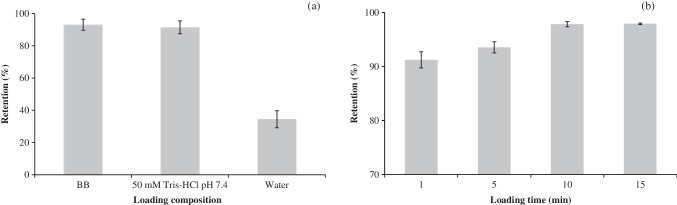
Influence of loading solution (**a**) and extraction time (**b**) on the on α-LA retention using the Apt@cit-VMNPs. Error bars show the standard deviation of the results (n = 3). Experimental conditions (except the parameter under investigation): volume, 2 mL of 1 µg·mL^−1^ α-LA; extraction time, 5 min; stirring at 900 rpm and 25 ºC

In order to obtain the best extraction performance of α-LA using Apt@cit-VMNPs, several parameters that affect both the loading and elution steps were investigated. For each parameter tested, three replicates were conducted. The first parameter investigated was the influence of the loading solution composition. For this purpose, 25 mg of Apt@cit-VMNPs were mixed with 2 mL of 1 µg·mL^−1^ α-LA solution prepared in BB (Sect. "Reagents and materials"), 50 mM Tris–HCl at pH 7.4, and water. In all cases, the suspension was shaken at 900 rpm for 5 min at 25 °C. As shown in Fig. 3a, similar extraction efficiencies were obtained with BB and with 50 mM Tris–HCl buffer (93 ± 3 and 91 ± 4%, respectively). Therefore, the selected buffer for further studies was 50 mM Tris–HCl at pH 7.4, as it offers a simpler composition and aligns with the buffer used for the extraction of proteins from food samples.

The next parameter evaluated was the loading time. Different times (comprised between 1 and 15 min) were tried, while keeping constant the other loading conditions (2 mL of 1 µg·mL^−1^ α-LA in 50 mM Tris–HCl at pH 7.4, 900 rpm and 25 °C). As shown in Fig. 3b, the retention percentage ranged from 91.2 ± 1.5 to 97.91 ± 0.14%, reaching a plateau at 10 min. Consequently, this loading time was selected for subsequent experiments.

Before proceeding with elution optimization, the selected MSPE loading conditions were also tested with VMNPs and cit-VMNPs to assess the extent of binding between target analyte and the corresponding nanomaterial before the Apt attachment. An α-LA retention of 100% was obtained when VMNPs were used, whereas a retention of ca. 10% was obtained for the cit-VMNPs, which demonstrated the effective blocking of VMNPs using trisodium citrate. The poor retention of α-LA observed on cit-VMNPs could be due to the fact that, when the nanoparticle surface is blocked with citrate, both α-LA and the nanoparticles become negatively charged at pH 7.4, which generates electrostatic repulsion between them and significantly reduces non-specific retention. This effective suppression of nonspecific interactions ensures that, after Apt immobilization, α-LA binding onto Apt@cit-VMNPs is predominantly driven by specific molecular recognition. The immobilized Apt provides selective binding sites that recognize the target protein through a combination of hydrogen bonding, electrostatic interactions, and van der Waals forces, leading to the formation of a stable aptamer–protein complex.

**Fig. 4:**
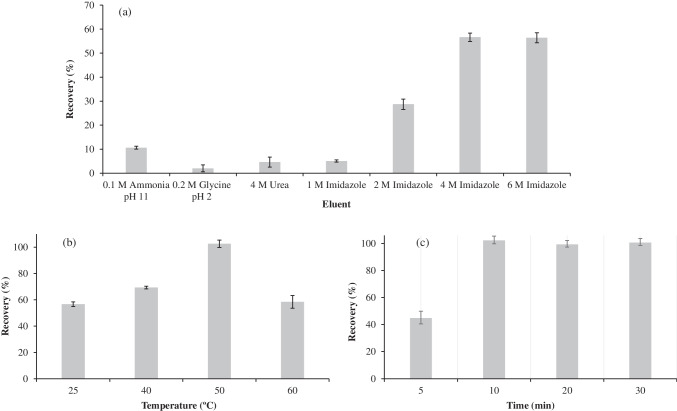
Influence of eluent (**a**), elution temperature (**b**) and elution time (**c**) on the α-LA recovery using the Apt@cit-VMNPs. Error bars show the standard deviation of the results (n = 3). Experimental conditions (except the parameter under investigation): volume, 2 mL of eluent; elution time, 10 min; stirring at 900 rpm and 25 ºC

Next, different elution conditions were investigated. It has been previously reported that protein elution from Apt-based sorbents can be achieved by changing temperature, ionic strength, or pH as well as by adding chelating or chaotropic agents [12, 33, 34]. Based on these strategies, and considering both our previous studies [20, 35] and other literature reports [34, 36], several eluents were tested (Fig. 4a). In all cases, 2 mL of eluent was used, and the suspension was shaken at 900 rpm for 10 min at 25 °C. Among the different eluents tested, the first candidates tried were 0.1 M ammonia at pH 11 [35] and 0.2 M glycine at pH 2 [20]; however, neither of these conditions provided good recovery values. Consequently, eluents such as 4 M urea [36] and 1 M imidazole [34] were assessed. As observed, these eluents also resulted in low recoveries, suggesting the need for stronger competitive displacement. A clear improvement in recovery was observed as the imidazole content increased from 1 to 4 M, with values rising from 5.1 ± 0.5% to 57 ± 2%, respectively. Increasing the imidazole concentration up to 6 M did not result in further improvement, with recovery reaching a plateau (56 ± 2%); therefore, a 4 M imidazole was selected for subsequent studies. The explanation for the results obtained when using imidazole as eluent is not straightforward, as they likely arise from the combined action of several mechanisms that collectively disrupt Apt-α-LA binding. At high concentrations, imidazole strongly competes with π-stacking and hydrogen-bonding interactions both within the Apt and at the protein–Apt interface. Also, the basic environment generated by imidazole increases the negative charge of α-LA, intensifying electrostatic repulsion with the negatively charged phosphate backbone of the Apt. In addition, this eluent can act as a mild chaotropic agent, perturbing the Apt’s tertiary structure, resulting in a loss of affinity for the target protein. In any case, these combined effects greatly reduce the Apt’s affinity for a-LA, making high imidazole contents (at 4 and 6 M) effective eluting agents for releasing this protein from the aptamer matrix.

Next, in order to improve α-LA recovery, different elution temperatures were tried. The results obtained are shown in Fig. 4b. As observed, recovery increased when temperature was increased from 25 to 50 ºC, reaching a maximum of 103 ± 3%. This result could be attributed to the fact that rising the temperature to 50 ºC favours mass-transfer kinetics and slightly relaxes the Apt’s tertiary structure without compromising its functional fold, thus facilitating protein release without significantly affecting α-LA integrity. In contrast, increasing the temperature to 60 ºC produced a relevant decrease in recovery, which could be attributed to more pronounced conformational perturbations in the Apt, diminishing its ability to undergo reversible structural transitions during elution. Therefore, 50 ºC was selected as the optimal elution temperature. Also, different elution times, comprised between 5 and 30 min, were tested (see Fig. 4c). At 5 min, recovery was 45 ± 2%, which was largely increased at 10 min (103 ± 3%), after which no further improvement was observed. Therefore, 10 min was selected as optimal elution time.

Additionally, different elution volumes (from 0.25 to 2 mL) were evaluated (lower volumes were not considered since at least 0.1 mL was needed for HPLC injection). A decrease in recovery values (< 70%) was found when 0.25 or 0.5 mL were used, while 1 mL of eluent provided recoveries comparable to those obtained with 2 mL (ca. 100%) (see Fig. S4). Therefore, 1 mL was selected, since it ensured the largest preconcentration factor (PF = 2) without compromising satisfactory recovery.

### Selectivity of Apt@cit-VMNPs

To evaluate the selectivity of the prepared Apt@cit-VMNPs, other milk allergenic proteins that can be present in the selected food samples (such as BSA, LF, α-casein, and β-LG) were subjected to the optimized MSPE protocol. Therefore, standard solutions of these proteins (at 1 μg·mL^−1^) were prepared in 50 mM Tris–HCl pH 7.4, being their recovery evaluated by HPLC after MSPE. As shown in Fig. S5, recoveries were below 5% for LF and β-LG, and less than 1% for BSA and α-casein. These results clearly demonstrated the high selectivity of the developed Apt@cit-VMNPs towards α-LA.

### Analytical performance

Different analytical parameters and figures of merit of the optimized Apt-MSPE method were evaluated. First of all, a good linearity (r > 0.999, Y = 29.837C_α-LA_ – 10.221, where Y = peak area and C_α-LA_ = α-LA concentration expressed as μg mL^−1^) was obtained in a concentration range comprised between 0.25 and 25 μg·mL^−1^ α-LA. The instrumental LOD and LOQ values were determined at signal-to-noise ratios of 3 and 10, respectively. Thus, taking into account the PF of 2, the LOD and LOQ values after Apt-MSPE method were 0.04 and 0.14 μg·mL^−1^.

Furthermore, the reproducibility of Apt@cit-VMNPs synthesis was evaluated by calculating the RSD of α-LA recoveries obtained at 1 μg·mL^−1^. The intra-batch reproducibility was established using Apt@cit-VMNPs obtained from the same synthesis, while the inter-batch reproducibility was assessed using materials prepared in different syntheses. The corresponding RSD values were 3.3 and 5.4% (n = 3), respectively, which demonstrates a satisfactory reproducibility of the preparation process. In addition, several extractions were performed using Apt@cit-VMNPs obtained from different batches after one month of storage in 50 mM Tris buffer at pH 7.4 at 4 ºC. In this case, an RSD value of 7% (n = 6) was obtained, which demonstrated that the sorbent remains stable for at least 1 month.

**Fig. 5:**
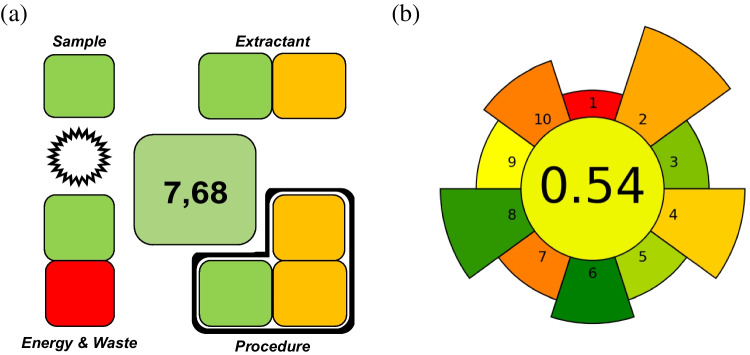
Comparison of the results obtained using different metrics applied to the proposed MSPE protocol: SPMS (**a**) and AGREEprep (**b**)

On the other hand, the sustainability of the method was assessed by employing two different metrics: the Sample Preparation Metrics of Sustainability (SPMS) [37] and the AGREE for sample preparation (AGREEprep) [38]. The final scores obtained (Fig. 5) were 7.68 and 0.54, respectively, which demonstrate the eco-friendliness of the developed method. It should be noted, however, that certain aspects of the protocol may be viewed as less advantageous, such as the off-line measurements, the need of heating in the elution step, the use of imidazole as eluent, and the multiple sample pretreatment steps common of the MSPE methods. Nevertheless, some of these limitations can be mitigated: the procedure enables the simultaneous processing of multiple samples, and the preconcentration step significantly reduces the amount of imidazole needed, contributing to a more sustainable overall approach.

### Determination of α-LA in food samples

**Fig. 6:**
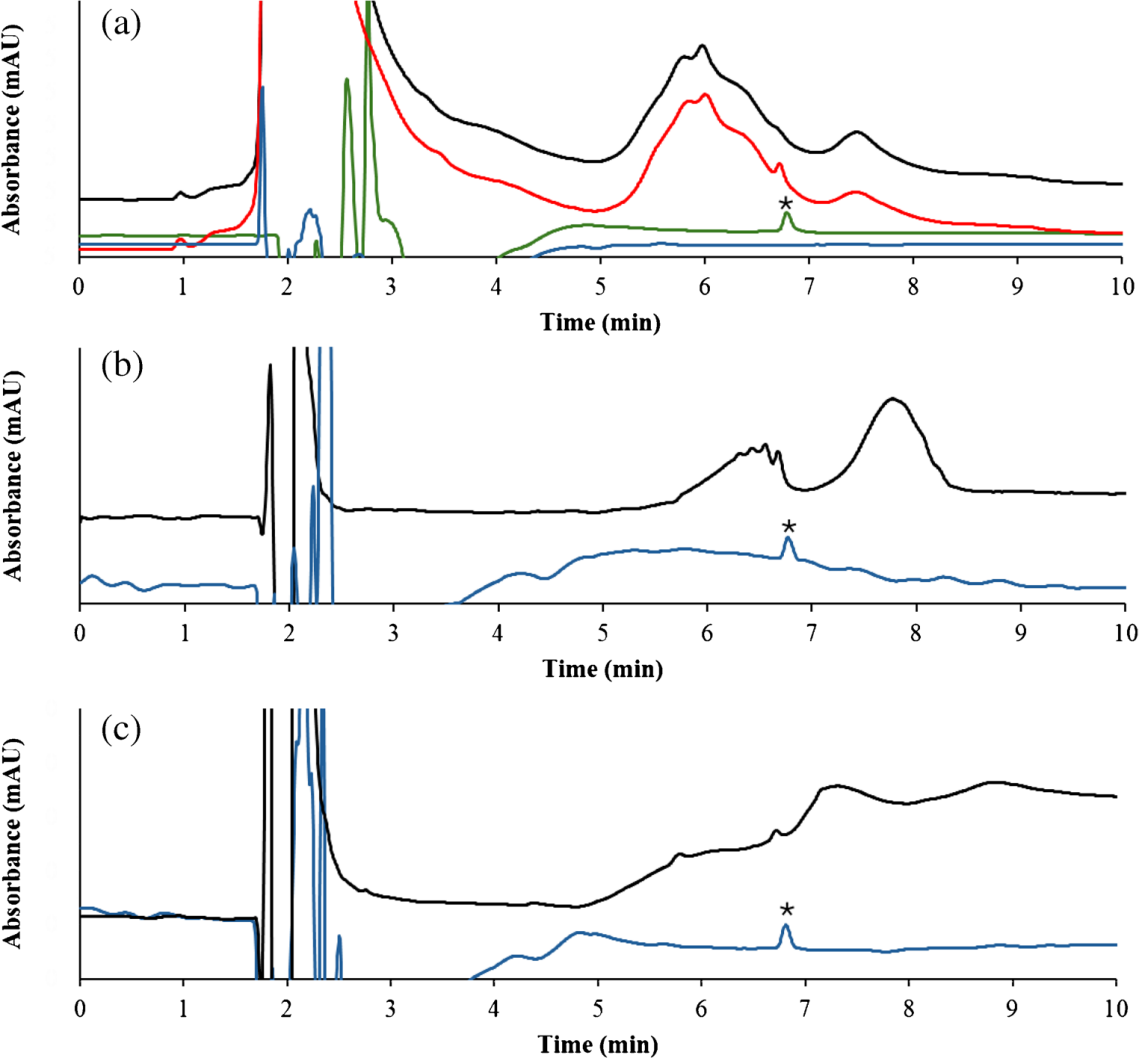
HPLC–DAD chromatogram before and after extraction procedure of α-LA in biscuit (a), infant formula (b) and milk chocolate (c). The black and red lines represent the non-spiked and spiked extracts before MSPE protocol, whereas the blue and green lines represent the elution of the non-spiked and spiked sample after MSPE protocol. Peak labelled with an asterisk corresponds to α-LA

The optimized MSPE method was applied to the determination of α-LA in several food samples (see Sect. "Reagents and materials"). In this sense, two dairy-free food samples (hydrolyzed hypoallergenic infant formula and sausages), one sample with declared milk trace levels (biscuits), and two samples with label declared milk (infant formula and milk chocolate) were analyzed. In the case of the two dairy-free foods and biscuits, α-LA was not detected. Therefore, these three sample extracts were fortified at two different concentration levels: 0.4 and 1 µg·mL^−1^ of α-LA (equivalent to 4 and 10 mg·kg^−1^), which was a concentration lower than the threshold value able to cause allergy symptoms [39, 40]. Figure 6a showed the chromatogram of biscuit extract before (black line) and after (red line) α-LA fortification, both before subjection to MSPE protocol, whereas blue and green lines showed the chromatograms of non-spiked and spiked biscuit extracts after MSPE method. As observed, the MSPE protocol resulted in a significant improvement in sample clean-up. This is particularly relevant when compared to conventional HPLC analysis, where selectivity is achieved mainly during chromatographic separation after injection. In contrast, the use of aptamer-based MSPE enables selective discrimination of the target analyte at the sample preparation stage, prior to chromatographic analysis, which is a critical advantage when dealing with complex food matrices. Furthermore, recovery.

values shown in Table 1 were satisfactory, which were comprised between 80 and 96.9%.

**Table 1:** Recovery results of α-LA determination in different food samples after the developed MSPE protocol (standard deviation, SD, n = 3)

Sample	Spiked concentration (mg·kg^−1^)	Found α-LA ± SD (mg·kg^−1^)	Recovery ± SD (%)
Hypoallergenic formula	4	3.53 ± 0.06	90.3 ± 1.7
	10	9.42 ± 0.11	96.5 ± 1.1
Sausages	4	3.19 ± 0.03	80.0 ± 0.7
	10	8.93 ± 0.13	89.5 ± 1.3
Biscuits	4	3.61 ± 0.04	90.6 ± 1.0
	10	9.65 ± 0.14	96.9 ± 1.4
Infant formula	-	0.31 ± 0.02 mg·g^−1^	-
	1 mg·g^−1^	1.23 ± 0.03 mg·g^−1^	92 ± 3
Milk chocolate	-	7.52 ± 0.05 mg·g^−1^	-
	1 mg·g^−1^	8.40 ± 0.02 mg·g^−1^	88 ± 2

On the other hand, infant formula and milk chocolate were also subjected to the MSPE method. Figure 6b and Fig. 6c showed the chromatograms before (black line) and after (blue line) treatment with Apt@cit-VMNPs of these samples. As observed in Fig. 6b, the chromatogram of the infant formula prior to the MSPE method exhibited the presence of interfering peaks, which difficult α-LA determination. In the case of milk chocolate (Fig. 6c), the α-LA peak was almost negligible. However, after MSPE, the chromatograms showed a substantial reduction of interfering compounds jointly with a clear enhancement of α-LA peak. This confirms that Apt-based sorbent selectivity at the sample preparation level provides an effective clean-up that cannot be achieved by chromatographic separation alone, enabling reliable α-LA determination in complex matrices. The α-LA content found in these samples is indicated in Table 1. In addition, both sample extracts were spiked with 1 µg·mL^−1^ of α-LA, yielding satisfactory recoveries values.

Finally, the reusability of the Apt@cit-VMNPs was examined. To this end, the developed MSPE protocol was repeatedly applied for α-LA extraction from both a standard solution (at 1 µg·mL^−1^) and from spiked sample extracts at the concentration levels indicated in Table 1 within one week. As observed in Fig. S6, recoveries above 80% were achieved up to the 3rd reuse for both types of samples.

### Comparison with other published methodologies

The analytical features of the developed Apt-MSPE method presented in this work were compared with previously reported procedures for α-LA determination in similar matrices (Table S1**)**. Among the different articles included in this table, only the study by Liu et al. [2] reported the use of an Apt-based sorbent. Regarding extraction time, the one achieved by our method is considerably shorter than that reported in this article [2] (20 vs 60 min). Although the total analysis time (including also food extract preparation and instrumental analysis) is longer in our work, this is mainly due to the fact that we dealt with more complex matrices, which needed longer sample preparation steps before extraction with the sorbent. Compared with immunoassay-based methods [5, 42], the proposed approach offers a clear advantage in terms of total analysis time, since techniques such as sELISA and ICA require several hours of analysis and, in some cases, involve extensive sample preparation steps. In contrast, the MSPE-HPLC–UV method presented here enables α-LA determination with a short extraction time (20 min) and reduced overall sample preparation requirements. In terms of recovery, the values obtained with our method are comparable to those previously reported by the other authors [2, 5, 41, 42]. Although the LOD of the developed MSPE method is slightly higher [2, 5, 42] and similar [41] than those reported, it remains sufficiently low for real food-safety applications. Taken together, these results indicate that the proposed method constitutes a practical, selective, and competitive approach for the determination of α-LA in complex food samples.

## Conclusions

In this work, an Apt-based MSPE sorbent was developed for the selective isolation of α-LA from food samples. The proposed approach highlights the value of introducing molecular recognition at the sample preparation stage, enabling selective discrimination of the target protein prior to chromatographic analysis and thereby enhancing method robustness when dealing with highly interferent-rich samples. Despite its advantages, the proposed method presents some practical and operational limitations inherent to MSPE-based approaches. Beyond its application to α-LA, the modular design of the sorbent and the immobilization strategy employed could potentially be adapted to other protein allergens by simply changing the Apt sequence. Overall, this work contributes to the advancement of selective sample preparation strategies for allergen analysis and supports the development of more reliable and adaptable analytical tools for food quality control, particularly in complex food matrices.

## Supplementary Information

Below is the link to the electronic supplementary material.

Supplementary file1 (DOCX 1383 KB)

## Data Availability

The data that support the findings of this study are available from the corresponding author upon reasonable request.
