# Supplementary material for: Aptamer modified magnetic nanoparticles for the determination of the allergenic protein α-lactalbumin in food samples
Source: Mikrochim Acta. 2026 Mar 3;193(3):212. doi: 10.1007/s00604-026-07936-5 (PMC12957121; doi:10.1007/s00604-026-07936-5)
Supplement: Supplementary file 1 — Supplementary file1 (DOCX 1383 KB) [file 604_2026_7936_MOESM1_ESM.docx]

**Aptamer modified magnetic nanoparticles for the determination of the allergenic protein α-lactalbumin in food samples**

**Natalia Piqueras-García, Raúl Mínguez-Peláez, María Vergara-Barberán, José Manuel Herrero-Martínez^*^, María Jesús Lerma-García^*^**

*Department of Analytical Chemistry, University of Valencia, Avda. Vicent Andrés Estellés 19, 46100 Burjassot, Valencia, Spain*

*Corresponding author: María Jesús Lerma-García

Tel.: +34963544497

e-mail: [m.jesus.lerma@uv.es](mailto:m.jesus.lerma@uv.es)

José Manuel Herrero-Martínez

Tel.: +34963544062

e-mail: [jose.m.herrero@uv.es](mailto:jose.m.herrero@uv.es)

**Fig. S1** XRD patterns of MNPs (a), VMNPs (b) and Apt@cit-VMNPs (c)

**Fig. S2** Complete N₂ adsorption–desorption isotherms and the corresponding BET linear plots of MNPs (a) and Apt@cit-VMNPs (b)

**Fig. S3** XPS full survey spectra of MNPs (a) and Apt@cit-VMNPs (b) and high-resolution spectra of Fe 2p (c), O 1s (d), C 1s (e), Si 2p (f), N 1s (g) and P 2p (h)

**Fig. S4** Influence of elution volume on the α-LA recovery using the Apt@cit-VMNPs. Error bars show the standard deviation of the results (n = 3). Experimental conditions: loading volume, 2 mL; elution time, 10 min; stirring at 900 rpm and 25 ºC

**Fig. S5** Selectivity study of the Apt@cit-VMNPs against α-LA and different milk-related proteins. Error bars show the standard deviation of the results (n = 3)

**Fig. S6** Recovery values of α-LA standard solution and spiked sample extracts (both at 1 μg·mL^-1^) obtained over successive cycles with repeated use of the Apt@cit-VMNPs

**Table S1**. Comparison of the proposed Apt-based MSPE method with other α-LA determination methods in similar matrices.

| Method | Sorbent | Sample | Extraction time (min) | Total analysis time (min) | Recovery (%) | LOD (µg·mL^-1^) | Selectivity | Ref. |
| --- | --- | --- | --- | --- | --- | --- | --- | --- |
| HPLC-MS/MS | - | Infant formula | - | 31.5 | 97.5-98.7 | 0.05 | Yes | [41] |
| Colorimetric/  fluorescence | Apt@  BNQDs/CeO_2_ | Milk and infant formula powder | 60 | 80 | 95.81–115.37 | 0.0007 | Yes | [2] |
| ICA | mAbs | Raw cow's milk and recombined milk | - | 435 | 97.60–102.96 | 0.01 | - | [5] |
| sELISA | mAbs | Hypoallergenic formula, chocolate bar, UHT milk, yogurt, whey powder, biscuits and red wine | - | *ca.* 17 h | 77–132 | 0.00159 | Yes | [42] |
| MSPE-HPLC-UV | Apt@cit-VMNPs | Infant formula, dairy-free formula milk chocolate, sausage, biscuit | 20 | 105-165 | 80.0– 96.9 | 0.04 | Yes | This work |

Abbreviations: Apt, aptamer; BNQDs, boron nitride quantum dot; ICA, immunochromatographic assay; mAb, monoclonal antibody; MNPs, magnetic nanoparticles; MSPE, magnetic solid phase extraction; sELISA, sandwich ELISA
